# Research Progress on the Hydrogen Embrittlement Resistance Performance of High-Entropy Alloys

**DOI:** 10.3390/ma18122862

**Published:** 2025-06-17

**Authors:** Xiao Kong, Hui Jiang, Yuting Lv, Wenlong Xie, Shuoyi Lu, Dingfeng Xu

**Affiliations:** 1 School of Mechanical and Electronic Engineering, Shandong University of Science and Technology, Qingdao 266590, China; sd123123202310@163.com (X.K.);; 2 College of Mechanical and Electrical Engineering, Qingdao University, Qingdao 266071, China; 3 School of Materials Science and Engineering, Dalian University of Technology, Dalian 116031, China

**Keywords:** high-entropy alloy, hydrogen embrittlement, hydrogen embrittlement mechanism, alloy design

## Abstract

The concealment and delayed characteristics of hydrogen embrittlement (HE) pose significant challenges for the development of hydrogen-resistant materials. As a novel category of multi-principal-element alloys, high-entropy alloys (HEAs) have emerged as ideal candidates for the next generation of hydrogen-resistant alloys due to their unique design philosophy, which endows them with excellent mechanical properties, corrosion resistance, high-temperature stability, and hydrogen embrittlement resistance. In recent years, research on the hydrogen embrittlement resistance of HEAs has attracted extensive attention. This review systematically summarizes the hydrogen embrittlement mechanisms in both conventional alloys and HEAs, critically analyzes the contradictions and controversial issues in the current literature, proposes design strategies for hydrogen embrittlement-resistant HEAs, and discusses future research directions in this field.

## 1. Introduction

Prolonged exposure to hydrogen, particularly in concentrated and high-pressure forms, has been demonstrated to induce hydrogen embrittlement, a process characterized by the deterioration of metal materials. This sudden catastrophic failure has the potential to result in significant economic losses and production safety risks that cannot be underestimated. The advent of hydrogen energy and the concomitant growth in demand have given rise to the necessity for materials that exhibit enhanced resistance to hydrogen embrittlement. Consequently, the development of alloys that exhibit exceptional resistance to HE has garnered significant interest from researchers.

In recent years, the processing technologies for HEAs have undergone continuous innovation to accommodate diverse material characteristics and application requirements. Traditional methods, primarily vacuum arc melting (VAM), are being progressively replaced by additive manufacturing (AM) techniques. AM technologies afford superior control over microstructural evolution through meticulous adjustment of processing parameters, rendering them indispensable for fabricating intricate HEA components. Among these, selective laser melting (SLM), electron beam melting (EBM), and Laser Powder Bed Fusion (LPBF) stand out as pivotal AM approaches [1].

Practical processing cases demonstrate that 316 stainless steel [2] and 304 stainless steel [3] fabricated by laser additive manufacturing exhibit stronger resistance to hydrogen embrittlement compared with traditional processes. Additionally, the CoCrFeMnNi high-entropy alloy prepared via Laser Powder Bed Fusion (LPBF) technology also maintains excellent hydrogen embrittlement resistance [4]. Shiratori [5] used the LPBF process to fabricate CoCrFeNiTi-based high-entropy alloys, which showed superior strength and hydrogen embrittlement resistance to Inconel 718 alloy through corresponding heat treatment processes.

**Figure 1:**
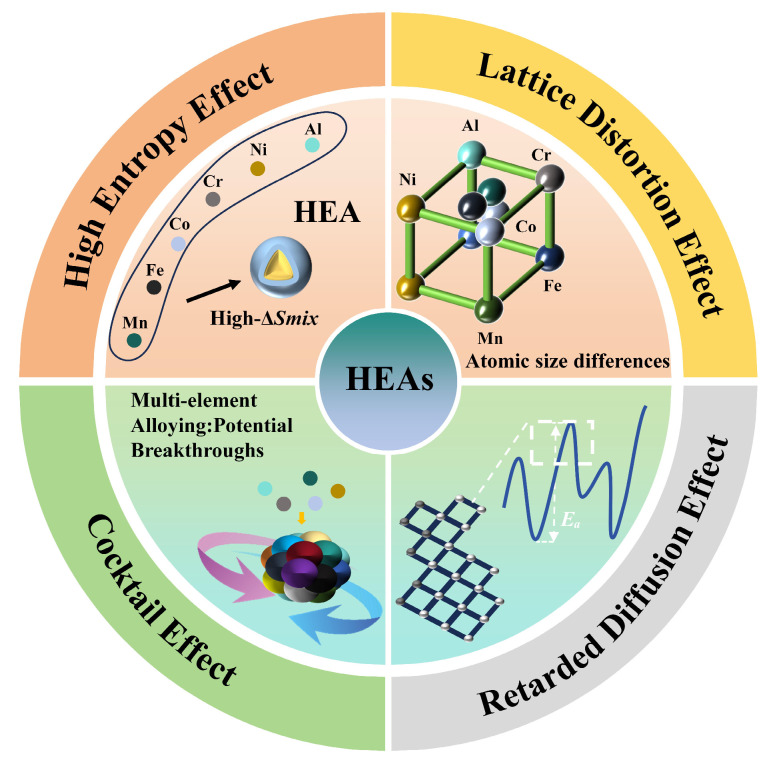
The four key effects of high-entropy alloys.

HEAs exhibit unique multi-principal-element compositions [6], which give rise to four key effects: the high-entropy effect, severe lattice distortion effect, sluggish diffusion effect, and cocktail effect [7]. The aforementioned effects are illustrated in Figure 1. The aforementioned effects engender a distinction between HEAs with microstructures and excellent properties and traditional alloys [8,9]. For instance, in certain single-phase face-centered cubic (FCC) [10,11], or body-centered cubic (BCC) [12] HEAs, hydrogen atoms demonstrate a reduced propensity for local accumulation, thereby exhibiting effective resistance to HE [13]. In comparison with single-phase FCC or BCC HEAs, eutectic HEAs with dual-phase or multi-phase structures exhibit remarkable mechanical properties [6]. In previous studies [10,14], the CoCrFeMnNi alloy has been the focus of extensive research in the presence of hydrogen. The majority of these studies have demonstrated the alloy’s notable resistance to hydrogen damage and its reduced hydrogen diffusion ability compared to austenitic stainless steel. This phenomenon can be attributed to the high barrier-energy characteristic of HEAs, which hinders the complete diffusion of hydrogen into the alloy, thereby exhibiting hydrogen insensitivity. Zhao [14] found through comparisons in a gaseous hydrogen environment that the hydrogen content in the HEA was higher than that in 316L stainless steel, yet no obvious HE occurred. This phenomenon was attributed to the high hydrogen solubility formed by its multi-principal-element composition and the low threshold of the hydrogen-enhanced localized plasticity (HELP) mechanism, suggesting that HEAs may exhibit greater potential for resisting HE in practical hydrogen environments. In their investigation of HE mechanisms in (FCC) medium-entropy alloys (MEAs) and stainless steels, Zhou [15] reported that the CoNiV MEA, despite sharing the same FCC structure, exhibits an exceptionally high critical hydrogen concentration threshold, making it highly resistant to embrittlement. However, a substantial body of research has revealed that the strength of the CoCrFeMnNi alloy typically ranges from 250 to 300 MPa. This finding indicates a need for the development of more advanced high-entropy alloys that can simultaneously enhance strength and toughness through process control and composition optimization.

**Figure 2:**
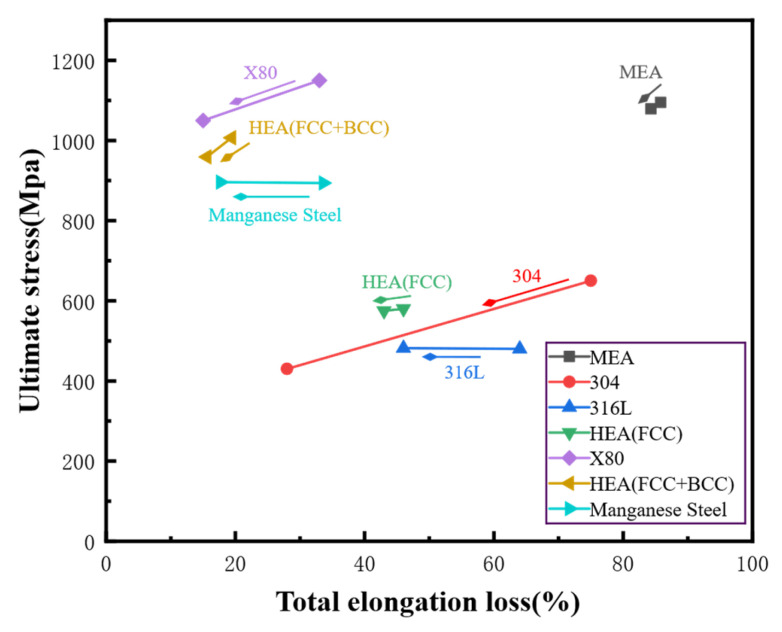
Comparison of hydrogen embrittlement resistance between HEAs and other metals at room temperature (the arrow direction represents the strain rate after hydrogen charging) [14,16,17,18].

In addition, it is broadly acknowledged that multi-component alloys exhibit more pronounced lattice distortion compared to conventional alloys and gold, which inherently possess a higher potential for application in the domain of hydrogen storage. Lattice distortion has been shown to cause instability in the interstitial sites that are occupied by hydrogen, thereby impeding the processes involved in hydrogen migration. Conversely, lattice distortion gives rise to the irregular diffusion path of hydrogen in high-entropy alloys, particularly when there is a significant change in the elemental composition. The cocktail effect itself will further exacerbate this diffusion effect. Figure 2 provides a synopsis of the mechanical property degradation of metallic materials currently being researched for their resistance to HE in hydrogen environments. As demonstrated, HEAs exhibit relatively less ductility loss and demonstrate excellent resistance to hydrogen embrittlement.

This article systematically reviews the mechanisms of HE in alloys and the research advancements in hydrogen embrittlement resistance of high-entropy alloys (HEAs). Addressing the core bottlenecks in current research, such as the ambiguous synergistic hydrogen resistance mechanisms of multi-principal elements and the lack of cross-scale predictive models for hydrogen embrittlement behavior, this study reveals the quantitative correlation among “lattice distortion degree—hydrogen trap density—high-energy trap hydrogen capture” in HEAs through integrating experimental data with theoretical simulations. This research also provides valuable insights into future research directions and deeply discusses the contradictions and controversies in current HE studies of HEAs through a comprehensive analysis. Finally, design strategies for developing HEAs with both high strength and high toughness against HE are proposed, aiming to promote further development in the field of HEAs.

## 2. Occurrence and Failure Modes of Hydrogen Embrittlement

### 2.1. Occurrence of HE

HE is defined as the phenomenon whereby metallic materials exhibit diminished toughness, degraded ductility, and even brittle fracture under specific environmental conditions due to hydrogen infiltration [19]. The causative factors of embrittlement can be categorized into two types: internal hydrogen and external hydrogen [20]. The presence of hydrogen within the material has been established as a pre-existing phenomenon, originating from various industrial processes such as smelting, pickling, electroplating, and welding. In the context of welded components, the presence of internal hydrogen has been demonstrated to be a contributing factor to hydrogen-induced delayed fracture. Conversely, external hydrogen is absorbed by the material during service [21], for instance, when hydrogen gas or hydrogen sulfide environments infiltrate the material [22]. The HE mechanism is closely related to the existence state of hydrogen in metals. Hydrogen can exist in metallic materials in a variety of forms, including H, H^+^, H^−^, H_2_, and metal hydrides. It is a commonly held view that hydrogen atoms remain in atomic form following their entry into a metal lattice [23].

HE is frequently observed to occur as a consequence of either excessive exposure in an acidic environment or the gradual adsorption of hydrogen onto the metal surface, whether physically or chemically, from an external environment. As a consequence of the process of diffusion, the presence of hydrogen species is known to be associated with crystalline imperfections. Such imperfections are understood to include lattice interstices, vacancy sites, structural defects, and grain boundaries. The result of this process is the eventual accumulation of hydrogen species at these locations, which can ultimately lead to the induction of hydrogen-related damage that may be either reversible or irreversible. The reversible damage phase is characterized by hydrogen-mediated plasticity reduction, a condition that can be mitigated through hydrogen source elimination coupled with subsequent desorption processes facilitated by temporal evolution or inert environment exposure. However, the engineering materials subjected to sustained hydrogen exposure have been demonstrated to exhibit heightened susceptibility to hydrogen-induced delayed cracking (HIDC), a failure mechanism associated with abrupt catastrophic fracture initiation and accelerated material degradation [19]. The development of critical hydrogen concentrations within the metallic microstructure can precipitate substantial internal pressure generation, which in turn, can lead to a range of irreversible damage modes. These include hydrogen-assisted cracking, flaking phenomena, and hydrogen blistering.

### 2.2. Overview of Hydrogen Traps

**Figure 3:**
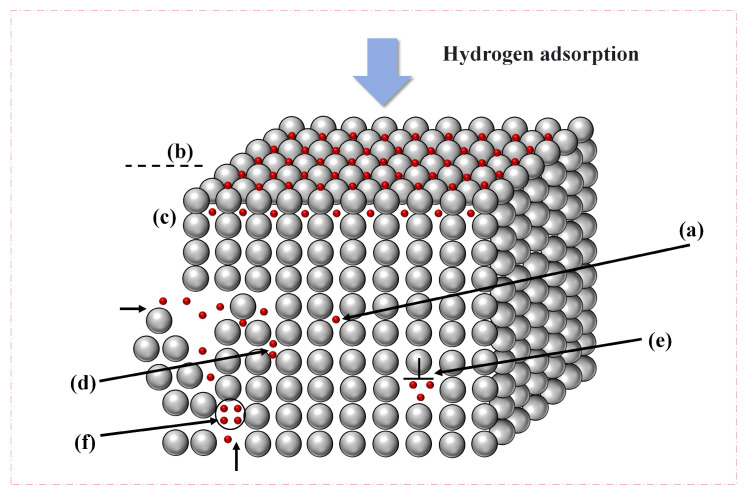
Hydrogen traps in the steels: (**a**) interstitial sites; (**b**) surface traps; (**c**) subsurface traps; (**d**) grain boundary traps; (**e**) dislocation traps; (**f**) vacancy traps.

Metals are known to exhibit various defects, including dislocations and vacancies, which are inherent to the material itself. In addition, void defects can result from differences in processing methods, while precipitates of second phases can be generated through microstructural control. In the process of permeation, hydrogen atoms are known to aggregate at defects in the metal matrix. Such defects are colloquially referred to as “hydrogen traps”. It is evident that the classification of these traps can be determined by the differences in binding energy; thus, they can be divided into two categories: reversible and irreversible hydrogen traps. As illustrated schematically in Figure 3, these hydrogen-enriched regions are defined as hydrogen traps. The trapping efficacy is found to govern three critical aspects of hydrogen–material interactions: namely, solubility thermodynamics, diffusion kinetics, and damage evolution mechanisms. Consequently, the strategic engineering of hydrogen trap characteristics (e.g., reversible or irreversible traps) is a pivotal methodology for enhancing material resistance against HE and stress-corrosion cracking. This is achieved by means of the following: type classification, real density, and spatial distribution. The prevailing classification systems categorize hydrogen traps by their binding energy magnitudes. Established threshold values are utilized to differentiate between distinct categories [23].

(1) The weakest traps (5–10 kJ/mol), represented by lattice interstices, primarily interact with diffusible hydrogen species that maintain high mobility within the crystalline structure.
(2) Weak traps (15–25 kJ/mol), encompassing low-angle grain boundaries, martensite lath interfaces, and dislocation networks, demonstrate intermediate hydrogen retention capacity for mobile hydrogen populations.
(3) Moderate-strength traps (30–50 kJ/mol), exemplified by microvoids and incipient cracks, effectively immobilize hydrogen through stronger interactions while permitting limited short-range diffusion.
(4) The strongest trapping sites (60–80 kJ/mol), particularly phase boundaries, completely arrest hydrogen mobility through high-energy interfacial interactions, resulting in permanent hydrogen sequestration [24,25].

### 2.3. Failure Mechanism of Hydrogen Embrittlement

In 1875, Johnson [26] was the first to report the phenomenon of hydrogen embrittlement. He observed that prolonged exposure of steel to hydrogen-containing media resulted in severe degradation of the steel’s mechanical properties, leading to brittle fracture. Notwithstanding the exhaustive research that has been conducted, the precise mechanism of HE remains to be fully elucidated. The following theories have been advanced to explain this phenomenon: the theory of hydrogen internal pressure, the theory of surface energy reduction, the theory of hydrogen-induced lattice decohesion, the theory of hydrogen-enhanced local plasticity, the theory of hydrogen-promoted strain-induced vacancy formation, and the theory of stress-induced hydride formation.

**Figure 4:**
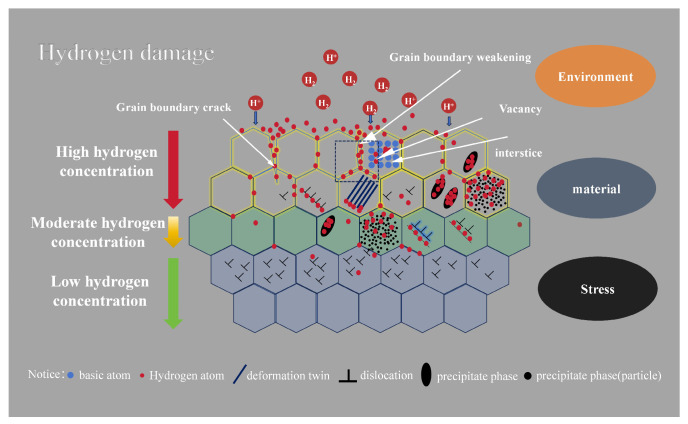
Hydrogen-induced damage mechanism schematic diagram.

Whilst four principal mechanisms currently dominate research on hydrogen embrittlement, ongoing mechanistic debates underscore the complexity inherent in hydrogen-material interactions. Industrial failure analyses typically reveal multifactorial origins involving the synergistic action of hydrogen-rich environments and mechanical stress states. Figure 4 presents a schematic representation of the mechanism of HE evolution under concurrent environmental exposure and stress loading. This mechanism delineates three pivotal stages: the initial entry of hydrogen into the material, the subsequent defect-facilitated accumulation, and the culmination in final crack propagation. This conceptual framework underscores the time-dependent nature of damage accumulation in hydrogen-induced failures, wherein opposing mechanisms may operate in parallel across diverse microstructural dimensions.

(1) Hydrogen Internal Pressure Theory

**Figure 5:**
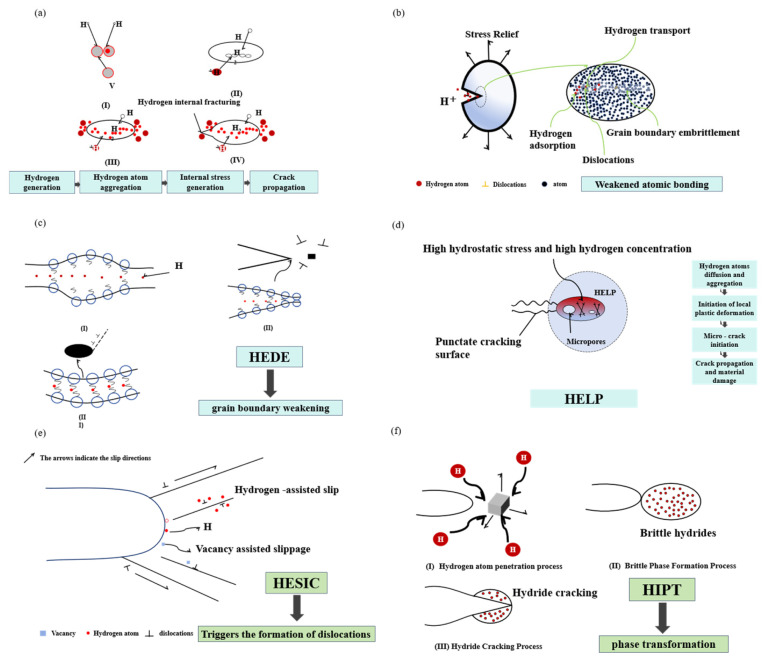
Hydrogen-induced failure mechanisms [24]: (**a**) HIPTM; (**b**) SERHEM; (**c**) HEDE; (**d**) HELP; (**e**) HESIC; (**f**) HIPT.

The hydrogen internal pressure theory posits that HE occurs as a consequence of pressurized hydrogen accumulation in metals [27,28]. As demonstrated in Figure 5a, hydrogen atoms diffuse and combine into hydrogen molecules within fixed metal gaps. It has been established that, upon reaching a critical level of hydrogen concentration, the subsequent build-up of gas pressure engenders internal stresses, thereby initiating the formation of cracks [29]. It is important to note that this mechanism provides a comprehensive explanation for hydrogen-induced cracking in the absence of external stress, particularly regarding the presence of hydrogen flakes in metal components and the delayed cracking that is frequently observed in welded structures following hydrogen absorption.

(2) Surface Energy Reduction HE Model

Petch [30] proposed the surface adsorption theory, suggesting that hydrogen dissolved in metal lattices preferentially accumulates at grain or phase boundaries. This hydrogen segregation alters atomic bonding states at interfaces, facilitating crack propagation as observed in hydrogen-charged materials. In high-strength steels and alloys, hydrogen accumulation weakens atomic cohesion at these boundaries. The non-uniform atomic configuration at grain boundaries further enhances hydrogen segregation [31], leading to a reduction in interfacial energy and rendering these regions favorable sites for crack nucleation and propagation, as depicted in Figure 5b.

(3) Hydrogen-Induced Lattice Weakening Mechanism

The Hydrogen-Enhanced Decohesion Mechanism (HEDE), initially proposed by Troiano [32] and later refined by Oriani [33,34], explains HE through electronic interactions. This theory suggests that hydrogen electrons reside in the d-electron bands of transition metals, where elevated electron density triggers interatomic repulsion. The resulting reduction in cohesive strength weakens atomic bonding, as depicted in Figure 5c. By unveiling the electronic underpinnings of hydrogen-induced failure, HEDE has emerged as a core framework for developing hydrogen-resistant materials, with notable validation in elucidating the HE mechanisms of HEAs.

(4) Hydrogen-Enhanced Local Plasticity Mechanism

The hydrogen-enhanced localized plasticity (HELP) mechanism, also known as hydrogen-assisted dislocation motion theory, focuses on hydrogen-dislocation interactions. This theory illuminates how hydrogen segregation at dislocations aids in solute cloud formation (Cottrell atmospheres), thereby simultaneously immobilizing dislocations to induce localized strengthening while boosting dislocation mobility and multiplication. This dual effect promotes strain localization and increased dislocation density, fundamentally explaining why hydrogen enhances localized plastic deformation [35,36,37], as shown in Figure 5d.

The HELP theory currently represents another widely accepted mechanism in the field of HE of HEAs. Lynch [38,39] studied the relationship between reversible hydrogen damage, strain rate, and temperature in steels. In situ scanning electron microscopy observations revealed that hydrogen atoms tend to accumulate at non-sessile dislocations, slip obstacles, or other elastic defects within the steel matrix, forming solute hydrogen atmospheres (Cottrell atmospheres). However, researchers remain divided on the HELP mechanism. While hydrogen can pin dislocations, it simultaneously promotes dislocation mobility, creating this paradoxical behavior.

(5) Hydrogen-Enhanced Strain-Induced Vacancy Formation Mechanism

To explain the fracture morphology of HE in materials, Maire [40] proposed the hydrogen-enhanced strain-induced vacancy formation mechanism. This theory suggests that hydrogen atom accumulation within the alloy facilitates the generation of vacancy defects and markedly speeds up vacancy coalescence, ultimately resulting in micropore formation. When the stability of the crack tip is disrupted, fracture occurs, just as it is illustrated in Figure 5e. This mechanism explains the microvoid phenomenon observed on the hydrogen-charged tensile fracture surface. Additionally, this mechanism is similar to the HELP mechanism, both of which are associated with the Cottrell atmosphere [41]. The Cottrell atmosphere significantly influences the strength and plastic deformation of materials by pinning dislocations, hindering their movement. Especially during material deformation, dislocations must overcome the pinning effect of the Cottrell atmosphere to move, thereby increasing the yield strength of the material [42].

(6) Hydrogen-Induced Phase Transformation Theory

Westlake [43] established that stress-driven hydrogen redistribution in alloys leads to hydrogen supersaturation at crack tips, exceeding the critical concentration threshold required for the austenite to martensite transformation. This phase transformation-induced embrittlement mechanism creates preferential paths for crack propagation. Furthermore, the presence of hydride-forming elements (Ti, Zr, Hf, V, Nb, and Ta) introduces additional hydrogen trapping sites through metastable hydride precipitation. The cleavage fracture behavior exhibited by these hydrides under stress is indicative of a synergistic exacerbation of HE susceptibility. This is attributable to the combined phase transformation and brittle particle effects, as illustrated in Figure 5f.

**Figure 6:**
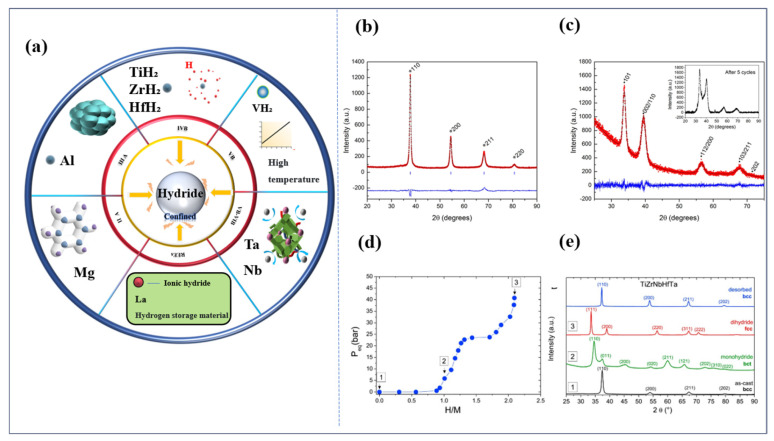
XRD phase transitions of TiVZrNbHf and TiZrNbHf [44,45]. (**a**) The following chart contains elements that are susceptible to hydride formation; (**b**) diffraction patterns of TiVZrNbHf as-synthesized (* indicates a single BCC phase) [44]; Reused from Ref. [44], open access. (**c**) diffraction patterns of TiVZrNbHf hydrogenated at 20 bar H2 and 400  °C for 48 h; peaks fitted in TOPAS (λ  =  1.540598 Å); the inset shows the diffraction pattern after the fifth hydrogen absorption (* indicates a single BCT phase) [44]; (**d**) the hydrogen absorption PCI curves for TiZrNbHfTa recorded at 300 °C [45]; (**e**) as-cast TiZrNbHfTa (black), monohydride (green), dihydride (red), and desorbed sample (blue) [45]. Reprinted from Ref. [45]. Copyright 2019, Elsevier.

The stability and transformation of the phase structure affecting high-entropy alloys are introduced. Firstly, it is demonstrated that the δ-atomic size mismatch and the valence electron concentration VCE are closely related. In high-entropy alloys, there is significant atomic size disparity in the atomic sizes of the multicomponent elements. This phenomenon is frequently accompanied by substantial distortions in the lattice structure. The presence of additional transition metals, predominantly those that are conventionally utilized as alloying elements in hydrogen storage applications, such as titanium (Ti), vanadium (V), zirconium (Zr), niobium (Nb), and hafnium (Hf), is indicative of the existence of these elements. Changes in phase structure stability and phase transitions are frequently observed phenomena. As demonstrated in the work of Sahlberg, M [44], the presence of larger δ values (e.g., δ > 6.8%) for TiVZrNbHf has been shown to promote the single-step phase transition BCC (B2) → FCC. Initially, the structure after complete hydrogenation was thought to be FCC; however, further analysis indicated BCT, suggesting that hydrogenation leads to a decrease in cubic symmetry and the formation of an intermediate phase structure. Following the hydrogen absorption–dehydrogenation cycle, while the BCC (B2) structure can be recovered, the peak shape splits, thereby demonstrating the reversibility of the phase transition process and the stability of the structure. In addition, Zlotea [45] investigated the hydrogen adsorption behavior of TiZrNbHfTa alloys with a BCC structure. After absorbing hydrogen, the HEA formed dihydrides with a face-centered cubic structure and generated monohydrides with a tetragonal structure in the intermediate phase. XRD provided substantiation for the alterations in the phase structure, as demonstrated in Figure 6b–d.

Existing theories regarding HE have been shown to have notable shortcomings when accounting for material-specific factors. Such factors include alloy composition and hydrogen charging methods. The failure of uniform theoretical models to explain the diverse range of HE phenomena is particularly problematic. Recent advancements in the field have brought to light an emerging consensus on synergistic multi-mechanism interactions that are the driving force behind embrittlement processes. In the field of HEAs, research into hydrogen compatibility remains in its infancy. The current body of knowledge attributes hydrogen-induced failures to two competing mechanisms. Assistance must be provided in conjunction with HEDE [46]. It is a curious paradox that certain experimental observations of premature cracking in pure hydrogen environments are more closely aligned with the hydrogen internal pressure theory. This suggests the possibility of a context-dependent dominance of different failure mechanisms, contingent upon specific stress states and hydrogen diffusion kinetics.

## 3. Research on HE Resistance of HEAs

### 3.1. Effects of Hydrogen on Mechanical Properties of High-Entropy Alloys

**Figure 7:**
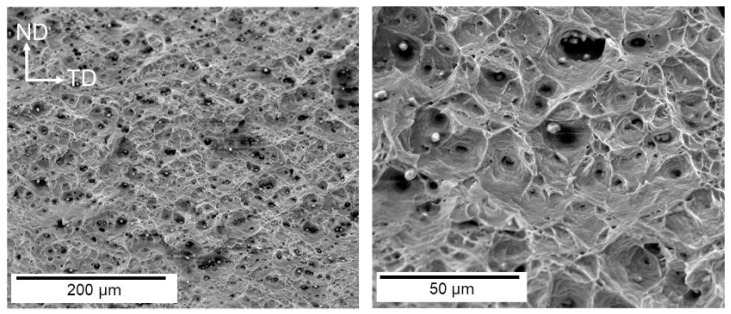
SEM characterization of the tensile fracture surface of unhydrogenated CoCrFeMnNi [48]. Reprinted from Ref. [48] Copyright 2024, Elsevier.

**Figure 8:**
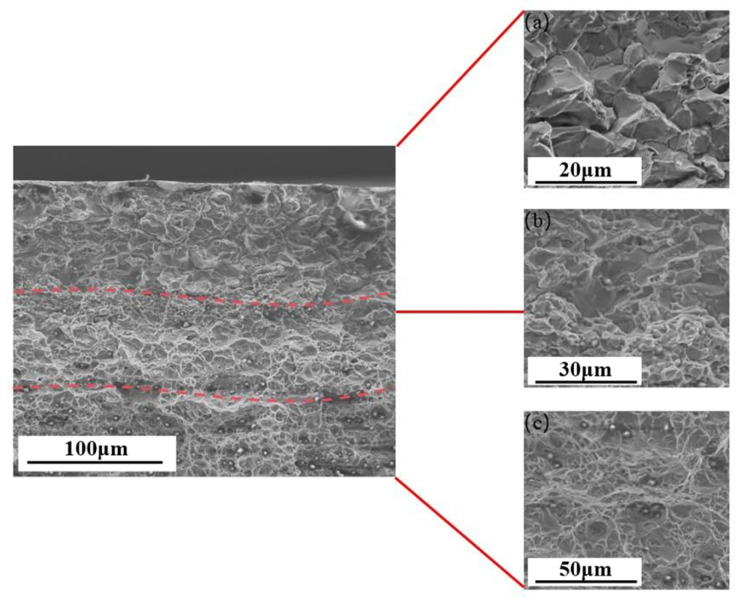
SEM Images of the fracture surface of hydrogen-precharged specimens and fracture surface images of different regions (The red dashed lines in the figure represent three different transition regions) [48]: (**a**) the fracture area near the surface; (**b**) the middle fracture area; (**c**) the internal fracture zone. Reprinted from Ref. [48] Copyright 2024, Elsevier.

The presence of the HE phenomenon is most commonly observed in high-strength steel, low-alloy steel, aluminum alloy steel, and martensitic stainless steel. Conversely, austenitic stainless steel is frequently employed as HE-resistant steel. At present, research on the HE behavior of HEAs is still in the preliminary exploration stage, with the majority of studies focusing on face-centered cubic (FCC)-structured CoCrFeMnNi, CoCrFeNi, and body-centered cubic (BCC)-structured FeCuCrMnMo and CoCrNi medium-entropy alloys. Li [47] discovered that the lattice distortion effect in HEAs hinders the stable diffusion of hydrogen atoms into the matrix. This results in a significantly higher hydrogen concentration on the alloy surface than in the interior after electrochemical hydrogen charging. The hydrogen concentration gradient gives rise to divergent fracture modes on the tensile fracture surface of the alloy, which can be categorized into brittle fracture zones, mixed fracture (quasi-cleavage fracture) regions, and ductile fracture areas. In a similar manner, Lisa [48] demonstrated that the fracture morphology of single FCC-structured CoCrFeMnNi HEAs following hydrogen charging is characterized by a hydrogen concentration-dependent evolution. As demonstrated in Figure 7, the fracture surface undergoes a sequential transition from intergranular fracture at the periphery to quasi-cleavage fracture in the intermediate region, culminating in ductile microvoid coalescence at the core. Intergranular fracture with distinct slip traces observed at the fracture edge (Figure 8a) aligns with the HEDE mechanism due to localized high hydrogen concentrations. The intermediate region exhibits quasi-cleavage features accompanied by residual ductile dimples (see Figure 8), suggesting that the deformation was dominated by the HELP mechanism. This phenomenon suggests that hydrogen charging alters dislocation motion pathways, promoting dislocation slip and thereby retaining partial ductile fracture characteristics.

**Figure 9:**
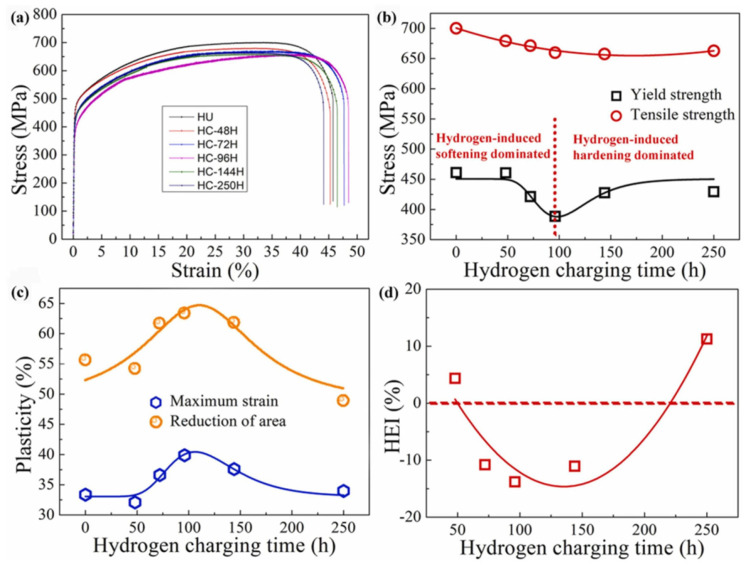
Mechanical property indexes of specimens with and without hydrogen charging under different durations [49]. (**a**) Engineering stress-strain curve; (**b**) plasticity; (**c**) HEI; (**d**) hydrogen charging time. Reprinted from Ref. [49]. Copyright 2022, Elsevier.

Li [49] investigated the effect of hydrogen charging time on the HE resistance of CoCrFeMnNi HEAs. As demonstrated in Figure 9a, the yield strength of the alloy exhibited an initial decrease, followed by an increase, with an increase in charging time. This non-monotonic property evolution is fundamentally attributable to the competition between the hydrogen-induced softening effect and the hydrogen-promoted twinning-induced strengthening effect. The results demonstrate that hydrogen charging induces nonlinear changes in the yield strength of FCC-structured HEAs. Figure 9b–d provide further elucidation on the trends in hydrogen-induced mechanical property variations, with the HE index (HEI) serving as a critical metric for quantifying the influence of hydrogen on mechanical behavior and characterizing the susceptibility to hydrogen embrittlement. The precise mathematical definition of HEI is given in Equation (1).

In Figure 9d, the following HE index (*HEI*) equation is used [49]:

(1) $$HEI=\frac{\varphi_{HU}-\varphi_{HC}}{\varphi_{HU}}\times 100\%$$

**Figure 10:**
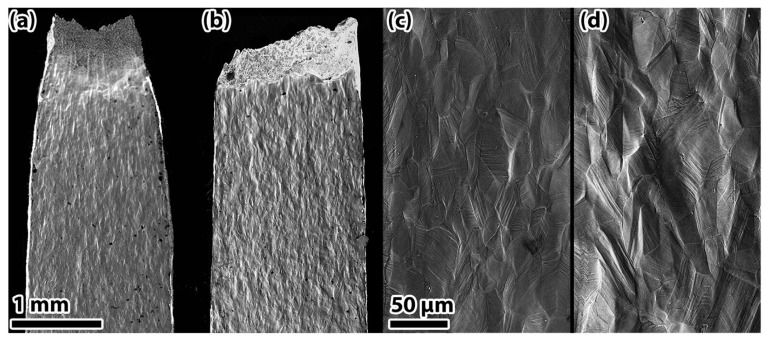
Longitudinal section of uncharged (**a**–**c**) and charged (**b**–**d**) FeNiCoCr [51]. Reprinted from Ref. [51]. Copyright 2018, Elsevier.

**Figure 11:**
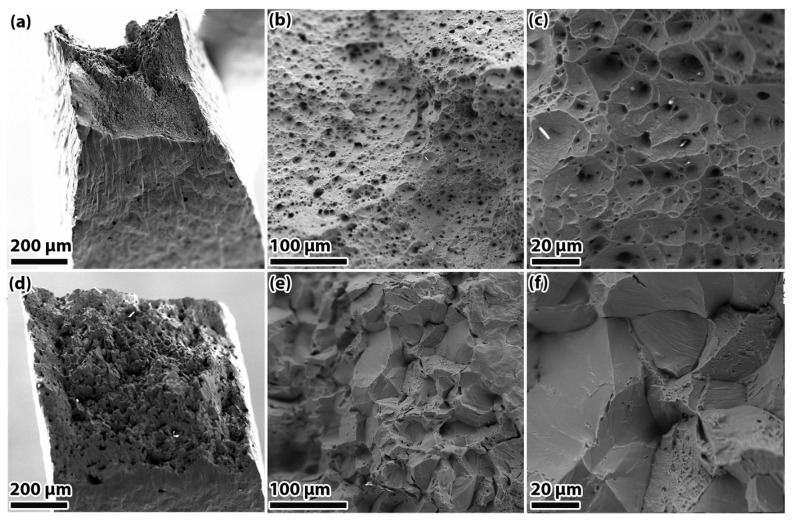
Fractographs of uncharged (**a**–**c**) and charged (**d**–**f**) FeNiCoCr [51]. Reprinted from Ref. [51]. Copyright 2018, Elsevier.

The (FeCoNi_86_)Al_7_Ti_7_ HEA predominantly exhibits hydrogen-induced intergranular cracking [50]. The initiation of crack formation is primarily observed at the grain boundaries, a phenomenon that can be attributed to the weakening of interfacial bonding strength due to the presence of hydrogen. In circumstances of applied stress, the presence of disordered atomic arrangements at L1_2_-phase grain boundaries has been demonstrated to exacerbate hydrogen segregation. This, in turn, has been shown to induce HEDE-driven failure behavior. In a similar vein, Kelly E. Nygren’s [51] study on the HE behavior of FeNiCoCr HEA revealed that hydrogen charging slightly reduced the material’s total elongation, with the fracture mode transitioning from transgranular ductile microvoid coalescence to intergranular fracture (Figure 10 and Figure 11). The absence of hydrogen has been shown to result in the absence of defects or embrittlement at the grain boundaries, and the material undergoes a transgranular ductile fracture through microvoid coalescence. Conversely, the propensity for intergranular fracture following hydrogen charging serves to further corroborate the notion that hydrogen exerts a substantial influence on grain boundary embrittlement.

**Figure 12:**
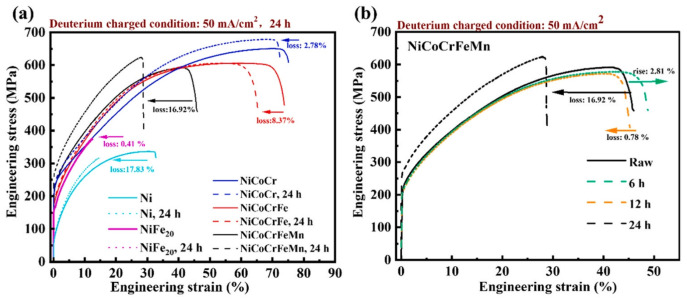
Stress–strain curves before and after electrochemical hydrogen charging [52]. (**a**) Stress and strain of four alloys after being loaded with deuterium for 24 h; (**b**) the stress and strain of NiCoCrFeMn under different deuterium charging times. Reprinted from Ref. [52]. Copyright 2024, Elsevier.

In a comparative study by An [52] on HE susceptibility of FCC-structured concentrated solid-solution alloys (CSAs), including NiFe_20_, NiCoCr, NiCoCrFe, NiCoCrFeMn, and pure Ni (Figure 12), distinct behaviors were observed. Deuterium charging reduced the tensile strength of pure Ni and NiFe_20_ alloys, whereas NiCoCr, NiCoCrFe, and NiCoCrFeMn alloys exhibited increased tensile strength. This enhancement in strength is attributed to deuterium atoms, which have been observed to promote dislocation accumulation and twinning growth through the reduction in stacking fault energy (SFE). This, in turn, has been shown to improve mechanical performance.

### 3.2. Microstructural Factors of HE in HEAs

#### 3.2.1. Effects of Alloying Elements

Alloying elements significantly influence HE susceptibility in metallic materials. Marques [53] investigated HE mechanisms in two HEAs: Fe_20_Mn_20_Ni_20_Co_20_Cr_20_ and Fe_22_Mn_40_Ni_30_Co_6_Cr_2_ with varying Cr content. Their results demonstrate that hydrogen diffusion coefficients decrease substantially with increasing Cr content, as Cr content governs hydrogen diffusion kinetics through lattice parameter modifications. Martin [54] revealed that controlled Cr additions (<20 wt.%) effectively enhance hydrogen embrittlement resistance while improving mechanical performance in hydrogen-containing environments.

An [52] conducted a comparative study on the HE behavior of alloys, including NiFe_20_, NiCoCr, and pure Ni, finding that Fe and Cr suppress deuterium solubility to mitigate embrittlement damage, while Mn increases solubility, leading to brittle fracture. Yi [55] demonstrated that the addition of Mo to a CoCrNi alloy enhances its resistance to hydrogen embrittlement. This is achieved by promoting twinning, which hinders hydrogen diffusion by restricting dislocation motion. Li [47,56] demonstrated that MoB phases induce twinning deformation and Al forms a passivation film, respectively, thereby enhancing the HE resistance of equiatomic CoCrFeNi HEAs. In the TiVZrNbHf high-entropy alloy (HEA), Ti synergizes with elements such as V, Zr, Nb, and Hf to form a multi-component solid solution, enabling hydrogen atoms to occupy not only the tetrahedral interstitial sites of traditional transition metal hydrides but also octahedral interstitial sites [44,45]. This unique feature is attributed to Ti’s high hydride formation energy (e.g., the formation energy of TiH_2_ is approximately−0.7 eV), which allows it to act as a reversible hydrogen trap—forming stable hydrides or solid solutions with hydrogen atoms to effectively reduce the concentration of free hydrogen in the lattice and inhibit hydrogen diffusion to crack tips. The robust hydrogen absorption/desorption properties of the TiVZrNbHf HEA endow it with significant application potential in hydrogen storage. However, it was also noted that HEAs generally exhibit a lower susceptibility to hydrogen embrittlement.

While different studies emphasize single mechanisms such as hydrogen solubility regulation, twin-mediated hydrogen diffusion inhibition, and surface passivation, hydrogen-induced failure in HEAs often results from multi-mechanism coupling—for example, the competition between grain boundary segregation and twinning-induced deformation. Current research has not fully elucidated the interactions between multi-scale defects (dislocations, twins, grain boundaries, and second phases) and hydrogen transport/accumulation. Consequently, studies that overemphasize the role of individual elements in isolation are overly simplistic and lead to fragmented mechanistic interpretations.

#### 3.2.2. Diffusible Hydrogen Content

The diffusible hydrogen content is an important factor in determining whether HE will occur in materials. HE occurs in materials only when the hydrogen content exceeds the critical level. The main factors that affect the hydrogen content are the method of hydrogen charging and the temperature.

At present, the mainstream hydrogen charging methods mainly include electrochemical hydrogen charging, gas-phase hydrogen charging, high-temperature and high-pressure hydrogen charging, and molten salt electrolysis hydrogen charging. Among them, electrochemical hydrogen charging can obtain the highest hydrogen content, with simple experimental requirements. And it is relatively easy to implement in the laboratory. In the electrochemical hydrogen charging experiment, the specimen to be charged with hydrogen is connected to the cathode of the power supply as the working electrode. While an inert electrode is connected to the anode of the power supply as the auxiliary electrode. When the circuit is turned on, the following chemical reactions will occur at the anode and cathode of the electrodes, respectively:

anode: 2H_2_O − 4e → 4H^+^ + O_2_

cathode: H + + e → H

2H^+^ → H_2_↑

overall reaction: 2H_2_O → 4H + O_2_

**Figure 13:**
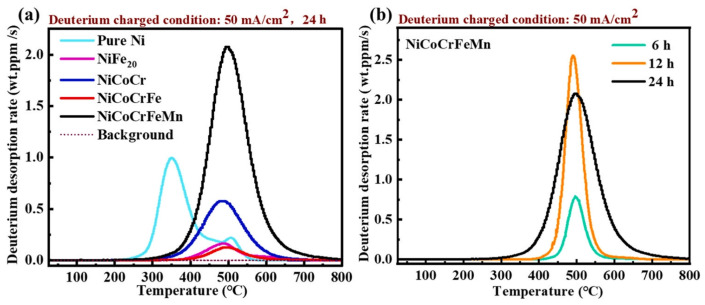
TDS test results of different samples [52]. (**a**) Different specimens with the same hydrogen charging time. (**b**) NiCoCrFeMn with different hydrogen charging times. Reprinted from Ref. [52]. Copyright 2024, Elsevier.

An [52,57] performed electrochemical deuterium charging on four multicomponent alloys and analyzed deuterium content via TDS (thermal desorption spectroscopy), with results presented in Figure 13a. The desorption spectra revealed two peaks in pure Ni: a low-temperature peak corresponding to reversible deuterium traps (typically associated with freely interstitial hydrogen from electrochemical charging), whereas alloys like NiFe_2_, NiCoCr, NiCoCrFe, and NiCoCrFeMn exhibited a single delayed desorption peak at approximately 500 °C, indicating the presence of deeper deuterium traps [12,17,58]. This behavior is attributed to the introduction of deuterium traps with higher binding energy, such as strong capture sites formed by solute atoms or defects via multi-element interactions, which significantly alter deuterium trapping dynamics [59,60]. Figure 13b further demonstrates a similar high-temperature desorption delay, corroborating the role of alloying in regulating hydrogen trap depth.

**Figure 14:**
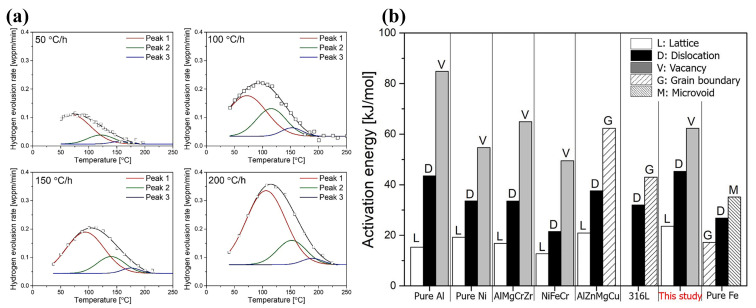
Decomposition results of the low-temperature peak of the sample [34,36,61,62,63,64,65,66]. (**a**) Lattice, dislocation, and vacancy [61]. (**b**) Review of activation energy values in the literature [34,36,62,63,64,65,66]. Reprinted from Ref. [52]. Copyright 2024, Elsevier.

Gao [61] revealed, through hydrogen microprint testing and TDS analysis, that diffusible hydrogen primarily accumulates at interstitial lattice sites (or cellular dislocations) rather than solidification cell walls or mechanical twins. The literature values of activation energy for diffusible hydrogen trapping sites show a distinct trend, with these values increasing sequentially for lattice sites, dislocations, and vacancy clusters. Considering both activation energy values and defect-type variations, it is reasonable to conclude that Peaks 1, 2, and 3 in Figure 14a correspond to lattice sites, dislocations, and vacancies, respectively, as illustrated in Figure 14b [34,36,62,63,64,65,66]. After pre-straining treatment, the primary trapping sites for diffusible hydrogen shift from as-cast distorted lattice sites to dislocations. These findings indicate that the effect of hydrogen on mechanical properties is primarily dependent on the total content of diffusible hydrogen rather than the type of hydrogen trap.

Table 1 presents a summary of deuterium atomic concentrations. Under consistent deuterium charging conditions, the NiCoCrFe alloy demonstrated the least amount of deuterium ingress. However, the incorporation of the Mn element has been shown to promote hydrogen absorption and diffusion. In this regard, the NiCoCrFeMn HEA with the highest deuterium concentration of 275.35 wt.ppm has been found to be closely related to the hydrogen (deuterium) charging current density.

#### 3.2.3. Hydrogen–Alloy Microstructure Interaction

The HE susceptibility of HEAs is highly dependent on their microstructure. Hydrogen typically accumulates near grain boundaries where cracks initiate, with hydrogen concentration varying according to grain boundary orientation [67,68]. Li [69] identified several key effects of twins on HE susceptibility. Twinning during deformation enhances HE resistance in CoCrFeMnNi alloys. Even at a hydrogen charging value of 53.6 wt.ppm, no embrittlement or hydrogen-assisted surface cracking occurred, attributed to nanoscale twin formation induced by hydrogen at 77K [70,71]. Luo [70] reported hydrogen-promoted twin formation in CoCrFeMnNi, simultaneously improving both HE resistance and strength. However, Sun [72] found that twins exerted marginal effects on the HE (HE) behavior of nickel. Deformation twins formed at crack tips during fatigue crack growth (FCG) testing of trapped hydrogen, increasing FCG rates in TWIP steel.

**Figure 15:**
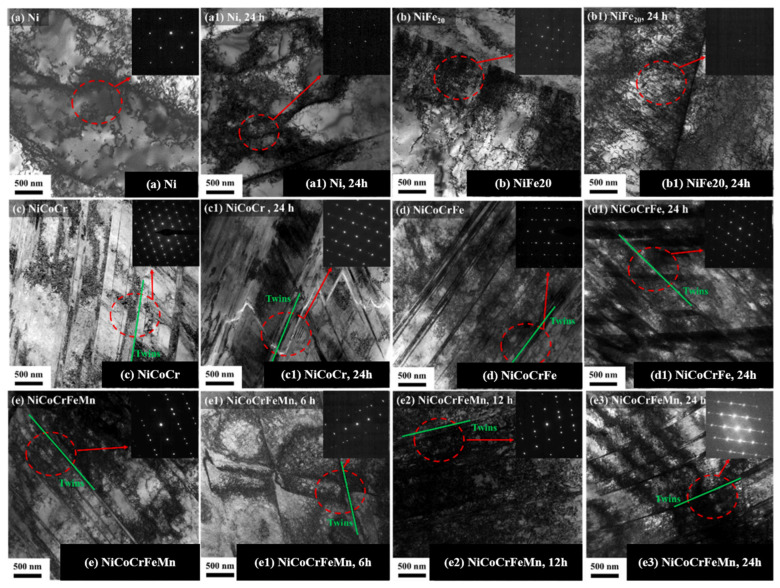
TEM results of Ni, NiFe_20_, NiCoCr, and NiCoCrFe. Samples after deuterium charging [52]: (**a**–**d**) original sample; (**a1**–**d1**) deuterium charging for 24h; (**e**) the original sample of NiCoCrFeMn; (**e1**–**e3**) NiCoCrFeMn samples are charged with deuterium for 6 h, 12 h, and 24 h. Reprinted from Ref. [52]. Copyright 2024, Elsevier.

An [52] conducted TEM investigations and observed extensive deformation twins during tensile testing of deuterium-charged NiCoCr, NiCoCrFe, and NiCoCrFeMn specimens (Figure 15). This phenomenon stems from deuterium-induced reduction in stacking fault energy [73,74]. Concurrently, deuterium atoms preferentially segregate near dislocations during deformation, promoting local work hardening [25,75,76].

**Figure 16:**
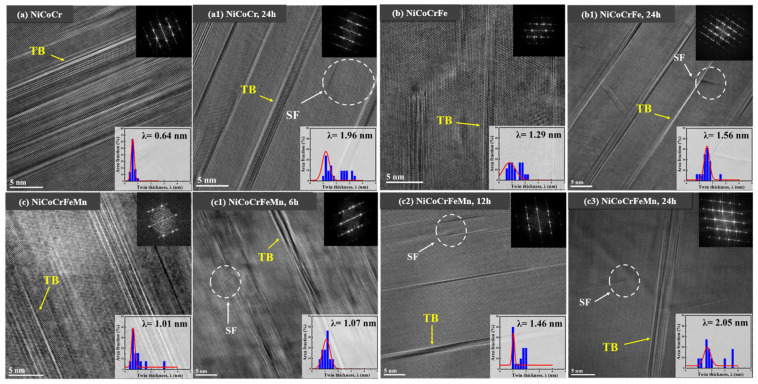
High-resolution TEM results of NiCoCr, NiCoCrFe, and NiCoCrFeMn samples [52]. (**a**–**c**) Original sample; (**a1**–**c3**) deuterium-charged for 6, 12, and 24 h. Reprinted from Ref. [52], Copyright 2024, Elsevier.

Selected area diffraction (SAD) patterns confirmed the twinning characteristics in the NiCoCr, NiCoCrFe, and NiCoCrFeMn alloys (Figure 16). Following deuterium charging, the mean twin thickness increased, suggesting that deuterium reduces the stacking fault energy (SFE). This SFE reduction promotes twin growth and elevates local stress, thereby facilitating HE [77,78] (Figure 16a1–c3).

**Figure 17:**
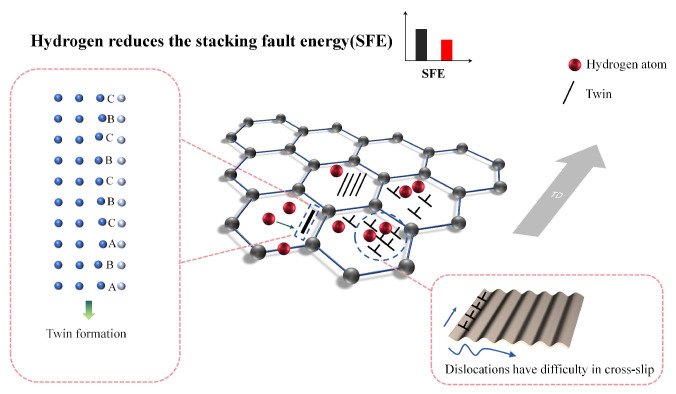
Schematic diagram of hydrogen reducing stacking fault energy.

The SFE is a critical parameter governing dislocation motion and deformation mechanisms in HEAs. Under static loading, the deformation of CrMnFeCoNi HEA is dominated by dislocation slip. However, hydrogen atoms alter this mechanism: the critical shear stress for twinning becomes lower than that for dislocation slip, making twinning the dominant deformation mode. As shown in Figure 17, hydrogen increases the dislocation expansion width, forming temporary dislocation pile-ups that impede dislocation movement along the original slip system. This necessitates non-slip deformation mechanisms (e.g., twinning) to coordinate strain, further suppressing dislocation slip dominance.

**Figure 18:**
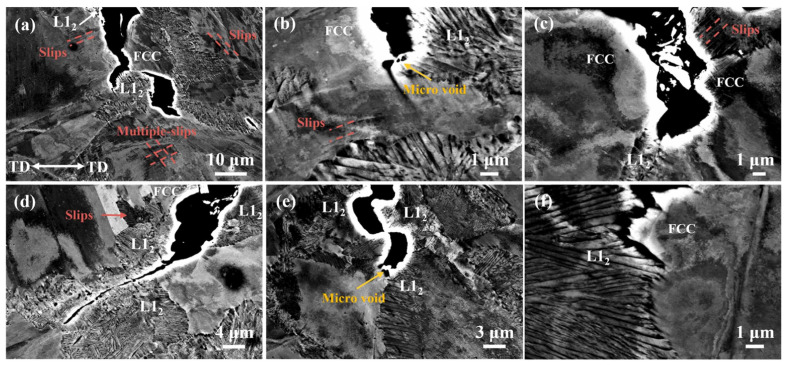
ECCI results of hydrogen-induced cracks on the side surface of Ni_50_Cr_20_Co_15_Al_10_V_5_ HEA after hydrogen-charged tensile testing [79]. (**a**–**f**) represent different shooting regions of the same sample. Reprinted from Ref. [79]. Copyright 2024, Elsevier.

Zhan [79] investigated the effect of the microstructure of precipitation-strengthened Ni_50_Cr_20_Co_15_Al_10_V_5_ HEA on hydrogen-induced cracking. This study revealed that hydrogen caused significant brittle fracture, with cracks propagating within the L1_2_ precipitate phase, as shown in Figure 18a. Analysis suggests that the cracking of L1_2_ precipitates originates from hydrogen-induced local concentration gradients. The FCC phase contains numerous dislocation slip bands, along with microvoids formed during hydrogen charging (located between the FCC and L1_2_ phases), as shown in Figure 18e. These microvoids likely serve as initiation sites for crack formation, with subsequent dislocation slip accelerating the propagation of intergranular cracks.

**Figure 19:**
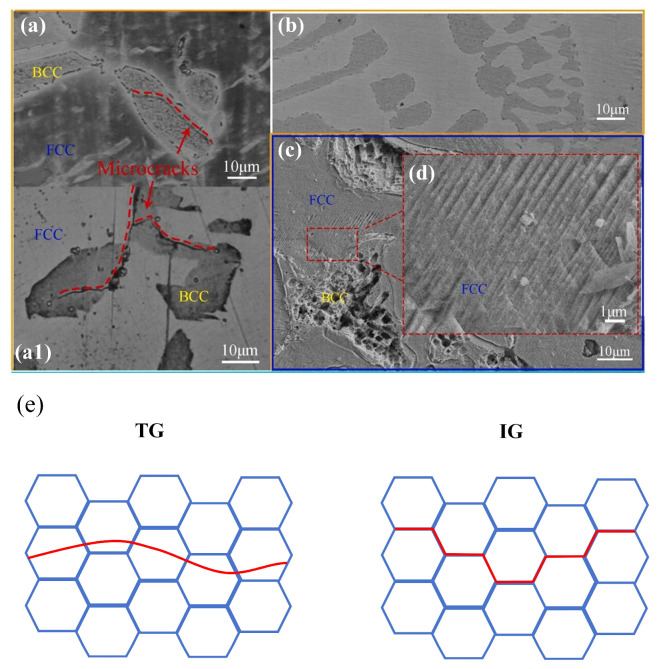
Surface changes of AlCoCrFeNi_2.1_ alloy due to electrochemical hydrogen charging [80]. (**a**) Cracks appeared after hydrogen charging for 9 h (**a**) and 24 h (**a1**). (**b**) Original morphology in the as-cast state. (**c**) Corrosion morphology after hydrogen charging. (**d**) is a high-magnification image of (**c**). (**e**) Transgranular cracking and intergranular cracking. Reprinted from Ref. [80]. Copyright 2023, Elsevier.

The phase boundary acts as a strong hydrogen trap, where hydrogen preferentially enriches, leading to hydrogen-induced damage at the interface. Feng [80] investigated the mechanical properties and fracture behavior of an FCC+B2 duplex AlCoCrFeNi_2.1_; equimolar high-entropy alloy (EHEA) after hydrogen charging, as shown in Figure 19a. Observations reveal that cracks preferentially initiate at the face-centered cubic (FCC)/body-centered cubic (B2) phase boundary following hydrogen exposure. The crystallographic orientation relationship of the phase boundary and the intergranular orientation difference significantly influence the formation of hydrogen-induced cracks [81,82]. Moreover, the B2 phase is more prone to hydrogen-induced damage compared to the FCC phase, as two distinct modes of hydrogen-induced cracking in the EHEA are illustrated in Figure 19d.

**Figure 20:**
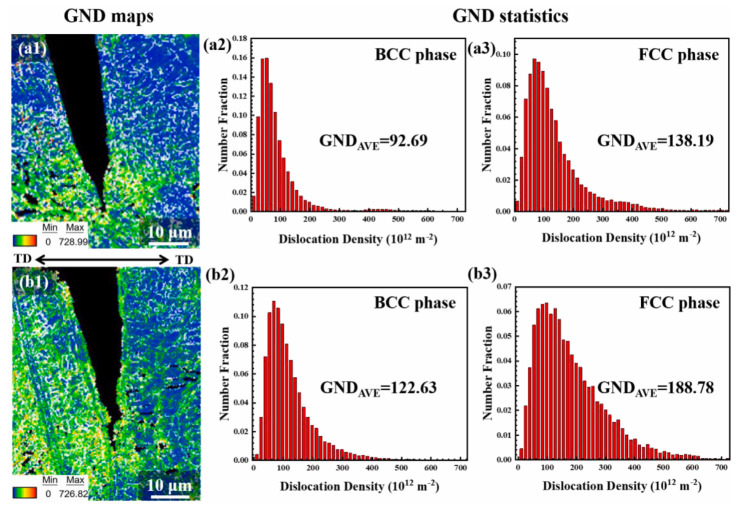
EBSD and GND density statistics of Co_30_Cr_10_Fe_10_Al_18_Ni_30_Mo_2_ after 48 h hydrogen charging and tensile test [75]. (**a1**,**b1**) GND maps of hydrogen-induced cracks; (**a2**,**a3**) and (**b2**,**b3**) correspond to the GND statistics of different phases of (**a1**,**b1**). Reprinted from Ref. [75]. Copyright 2024, Elsevier.

**Figure 21:**
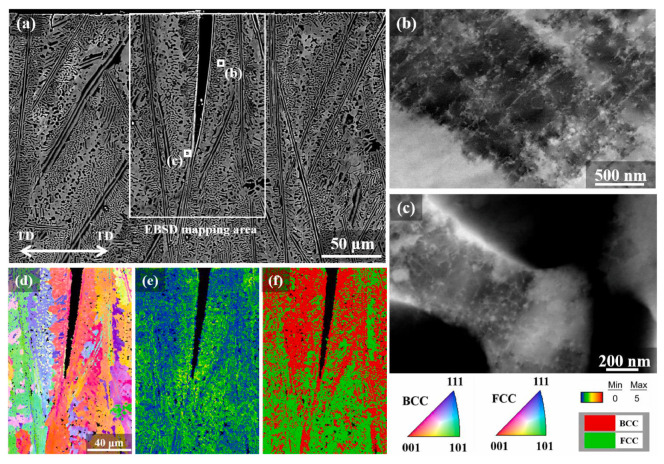
ECCI and EBSD analysis of cracks in Co_30_Cr_10_Fe_10_Al_18_Ni_30_Mo_2_ after 48 h hydrogen charging and tensile test [75]. (**a**) ECCI image of hydrogen-assisted crack; (**b**,**c**) locally enlarged areas shown; (**d**–**f**) EBSD surface distribution regions. (**d**) IPF map; (**e**) KAM map; (**f**) phase map obtained by EBSD. Reprinted from Ref. [75]. Copyright 2024, Elsevier.

Zhao [75] analyzed geometrically necessary dislocations (GNDs) at hydrogen-induced cracks and found crack arrest at highly strained FCC regions in Co_30_Cr_10_Fe_10_Al_18_Ni_30_Mo_2_ duplex EHEA (as shown in Figure 20). Using electron channeling contrast imaging (ECCI), another crack propagating from the sample edge inward was observed. Unlike Figure 20, this crack propagated through the BCC phase rather than along phase boundaries (Figure 21).

Grain boundary character distribution (GBCD) is closely correlated with hydrogen-induced cracking behavior, as high-angle grain boundaries (HAGBs) are prone to hydrogen enrichment due to their higher interfacial energy. In previous studies on pipeline steels, such as X70 steel (BCC structure), the significant absence of Σ29a-type CSL boundaries (46.3° <100> misorientation) in cracked specimens indicates their superior resistance to hydrogen-induced cracking (HIC) [83,84]. Notably, unlike the crack-inhibiting effect of Σ3 boundaries (60° <111>) in FCC materials (e.g., austenitic stainless steels), Σ3 boundaries in X70 steel do not exhibit crack resistance and may even serve as preferential cracking paths [84].

Recent research by Li et al. [85] has shown that, in AlCoCrFeNi_2.1_ eutectic high-entropy alloys (HEAs), hydrogen-induced cracking occurs more readily at high-angle grain boundaries (HAGBs) than at low-angle grain boundaries (LAGBs) or phase boundaries. The hydrogen concentration at HAGBs can reach 10–20 times that of the matrix, making them core sites for hydrogen-induced crack initiation [86]. In CoCrFeMnNi HEA, twin boundaries (e.g., Σ3) exhibit crack resistance similar to traditional FCC stainless steels, attributed to their low interfacial energy (~0.3 J/m^2^) and weaker hydrogen trapping capability compared to HAGBs [86]. This contrasts with the behavior in X70 steel. Further studies reveal that, in body-centered cubic (BCC) or eutectic (FCC+BCC) structures, Σ3 boundaries in BCC alloys may not form via twinning, leading to distinct crack resistance characteristics [87].

#### 3.2.4. Hydrogen Diffusion Behavior

The diffusion behavior of hydrogen in high-entropy alloys (HEAs) is governed by two primary factors. First, the HEAs exhibit sluggish diffusion inherent to their complex atomic structure, and second, lattice distortion effects disrupt lattice symmetry, leading to a non-uniform hydrogen distribution [85]. Hydrogen atoms preferentially occupy interstitial sites, a phenomenon attributed to their significantly smaller atomic radius. This structural feature exerts a profound effect on hydrogen diffusion pathways and rates, ultimately governing the overall diffusion process.

**Figure 22:**
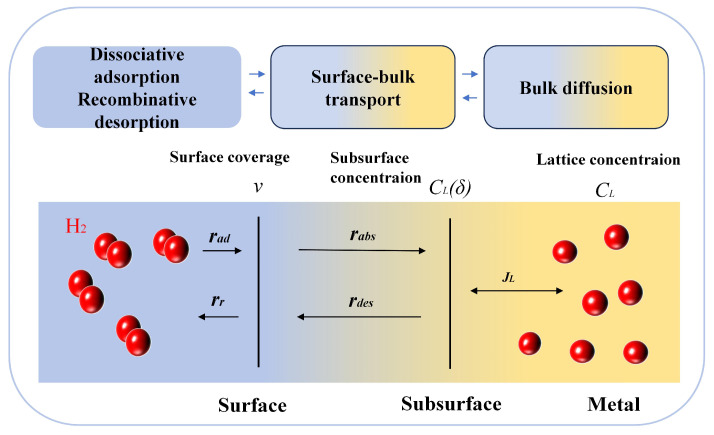
Schematic diagram of kinetic adsorption and absorption of hydrogen.

Figure 22 shows the kinetic analysis of hydrogen diffusion into metals, which involves dissociative adsorption (r_ad_), recombinative desorption (r_r_), absorption from the surface to the subsurface (r_abs_), and desorption from the subsurface to the surface (r_des_). The corresponding calculation formulas are as follows:

(2) $$r_{ad}=k_{ad}P_{H_{2}}{\left(1-\theta \right)}^{2}$$

(3) $$r_{r}=k_{r}\theta^{2}$$

(4) $$r_{abs}=k_{abs}\theta$$

(5) $$r_{des}=k_{des}C_{L\left(\delta \right)}$$

The rate of change of surface coverage *v* is as follows:

(6) $$\frac{d_{v}}{d_{t}}=r_{ad}-r_{r}-r_{abs}+r_{des}$$

Then,

(7) $$\frac{d_{v}}{d_{t}}=k_{ad}P_{H_{2}}{\left(1-v\right)}^{2}-k_{r}{v^{2}}_{r}-k_{abs}v+k_{des}C_{L\left(\delta \right)}$$

The rate of change of the subsurface concentration *C_L_ (δ)* is as follows:

(8) $$J_{L}=\frac{D_{L}\left(C_{L}-{C_{L}}_{\left(\delta \right)}\right)}{\delta }$$

(9) $$\frac{d_{CL\left(\delta \right)}}{d_{t}}=r_{abs}-r_{des}-J_{L}=k_{abs}v-k_{des}C_{L\left(\delta \right)}-\frac{D_{L}\left(C_{L}-C_{L\left(\delta \right)}\right)}{\delta }$$

Abbas Mohammadi [88] developed an integrated strategy to enhance HE resistance by selecting FCC-structured HEAs with inherently slow hydrogen lattice diffusion. This strategy involves selecting alloys containing elements that impede hydrogen transport from the surface to the bulk and introducing low-mobility lattice defects (e.g., nanotwins) via severe plastic deformation to suppress hydrogen migration. Specifically, controlled Al additions to CrFeCoNi alloy inhibit BCC phase formation, thereby further reducing hydrogen diffusivity. However, excessive Al content may precipitate B2 phases, which can paradoxically increase HE susceptibility, necessitating careful optimization of the alloy composition.

**Figure 23:**
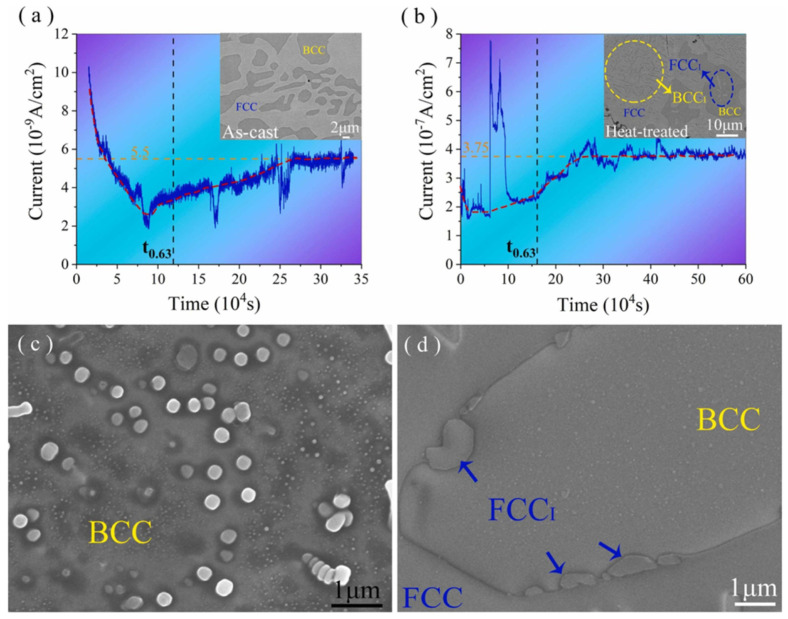
Electrochemical hydrogen permeation curve of AlCoCrFeNi_2.1_ [80]. (**a**) As-cast sample; (**b**) sample after heat treatment; (**c**) as-cast morphology; (**d**) morphology after heat treatment. Reprinted from Ref. [80]. Copyright 2023, Elsevier.

Claeys [48] investigated hydrogen interaction with CoCrFeMnNi HEA and found it exhibits higher hydrogen solubility and diffusivity compared to austenitic stainless steels, attributed to its complex chemistry enabling greater hydrogen accommodation and rapid diffusion pathways [81]. Feng [80] measured the hydrogen diffusion coefficients (D_hdc_) of as-cast and heat-treated AlCoCrFeNi_2.1_ EHEA, respectively. They are 1.09 × 10^−12^m^2^/s and 1.76 × 10^−14^m^2^/s, as shown in Figure 23a,b. These values were significantly lower than those of conventional metals and stainless steels.

**Figure 24:**
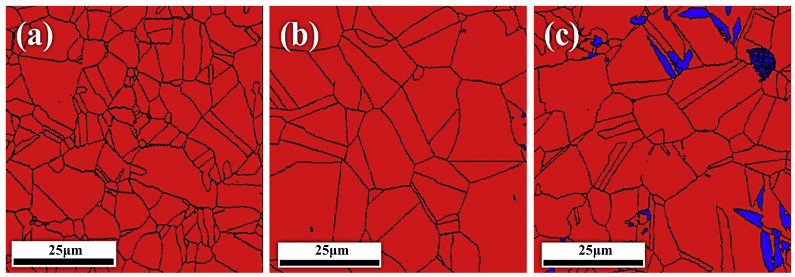
Microstructural behavior of various FCC metals after electrochemical hydrogen permeation testing [89]. (**a**) CoCrFeMnNi; (**b**) SS316L; (**c**) SS304. Reprinted from Ref. [89]. Copyright 2020, Elsevier.

Lee [89] first measured hydrogen diffusion coefficients of CoCrFeMnNi HEA and 304 and 316L stainless steels using electrochemical hydrogen permeation. Results showed comparable effective diffusion coefficients for CoCrFeMnNi HEA (1.81 × 10^−11^m^2^/s) and 316L SS (1.31 × 10⁻^11^ m^2^/s). After hydrogen permeation, HEA retained a lower hydrogen concentration (C_0_ = 0.043 mol/m^3^) compared to ferritic steel (C_0_ = 46.4 mol/m^3^) (as listed in Table 2). No microstructural changes occurred in HEA or SS316L, but martensite precipitation in SS304 led to further embrittlement (as shown in Figure 24).

It is hypothesized that computational models employed to simulate the HE mechanism have been effective in accounting for complex multi-major element effects in HEAs to a certain extent. This is primarily attributed to the differences in the interaction energies of the independent elements with hydrogen. Nevertheless, there are challenges in accurately capturing local lattice distortions and chemical inhomogeneities at the atomic scale. The ensuing analysis is derived from an examination of the model’s capabilities and limitations. Figure 7 offers a visual representation of the local chemical environment and the elemental interaction energies of hydrogen atoms in multi-major element alloys.

**Figure 25:**
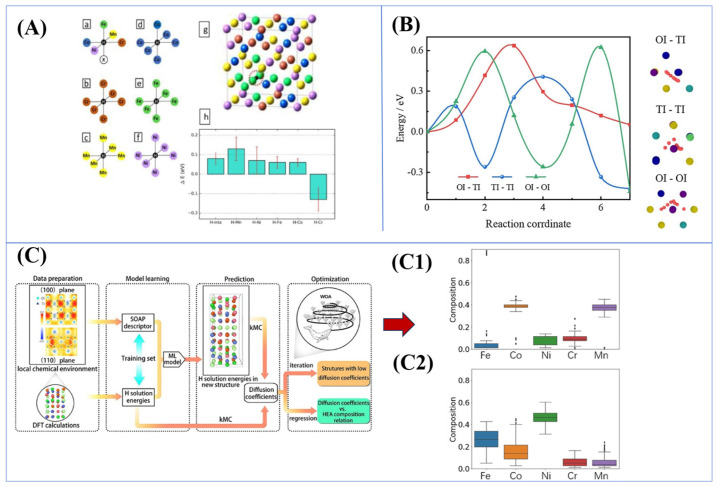
(**A**) Hydrogen atomic neighborhood for the six investigated systems. In a: H-mix, b: H-Cr, c: H-Mn, d: H-Co, e: H-Fe site, f: H-Ni sites, and g: example of a H-Fe configuration, the other H-Fe configurations also possess hydrogen surrounded by Fe, but the other atoms are differently distributed; h: the interaction energy promoted by H addition for H-Me pairs [53]. (**B**) Hydrogen diffusion pathways in HEAs, where red, blue, and green lines represent the diffusion paths of OI–TI, TI–TI, and OI–OI, respectively [90]. Reprinted from Refs. [53,90]. Copyright 2021, Elsevier. (**C**) Flowchart of the data preparation, model training, prediction, and optimization process for designing CoCrFeMnNi alloy [91]. (**C1**) Alloy elements with hydrogen diffusion coefficient < 10^−16^s^−1^. (**C2**) Alloy elements with hydrogen diffusion coefficient > 10^−14^s^−1^. Reprinted from Ref. [91]. Copyright 2022, Elsevier.

Firstly, first-principles calculations through density functional theory (DFT) are capable of quantitatively analyzing the differences in the interaction energies of different alloying elements with hydrogen. Sara Correa Marques et al. [53] calculated that the low interaction energy of Cr with hydrogen (~−0.21 eV) makes it a weak hydrogen trap, whereas the higher interaction energies of Co and Ni (~0.17–0.27 eV) tend to form strong traps. The observed phenomenon can be attributed to the potential presence of both Cr (weak traps) and Ni (strong traps) surrounding hydrogen atoms, resulting in a “gradient distribution” of local hydrogen concentration. This microscopic heterogeneity complicates the HE mechanism and makes it difficult to fully realize accurate simulations, as shown in Figure 25A. In a seminal study, Xie [90] employed first-principles calculations to investigate the solvation energies, diffusion paths, and potential barriers of hydrogen in tetrahedral and octahedral interstitials. Their meticulous analysis revealed a pronounced potential barrier and asymmetry in hydrogen migration, providing a comprehensive understanding of the fundamental processes involved. As demonstrated in Figure 25B, the range of diffusion barriers (0.17–1.05 eV) for the OI-TI pathway provides direct evidence that the migration resistance is significantly higher than that of pure metals. The diffusion hysteresis effect is a fundamental phenomenon that governs the mechanism under investigation. Theoretical simulations at the atomic scale provide a clear demonstration of this mechanism. These simulations reveal that increasing the diffusion barrier, breaking the symmetry, and enhancing the trapping effect can significantly reduce hydrogen mobility.

Zhou [91] employed a machine learning approach to design CoCrFeMnNi HEA with ultra-low hydrogen diffusion coefficients, as shown in Figure 25C. Their study characterized local chemical environments around H atoms in metal lattices [92] and then predicted H solution energy and diffusion coefficients of WOA-generated structures using ML models. Through iterative optimization, low-diffusion alloys rich in Co or Mn and HEAs rich in Fe or Ni were identified, as validated by simulation results from Figure 25C1,C2.

HE in HEAs is governed by the intricate interplay between hydrogen and microstructural features, especially in regions of stress concentration, where hydrogen impacts dislocation dynamics. Current research debates focus on whether hydrogen promotes [93,94,95], impedes [96,97], or has no effect [98,99] on dislocation evolution. For example, Wang [93] observed increased dislocation tangles in hydrogen-charged pure iron. Lawrence Cho [100] reported elevated dislocation density in hydrogen-charged high-Mn austenitic steel. However, Robertson [98] observed no significant changes in the dislocation structure and density of nickel after hydrogen charging, which contradicts earlier findings. In HEAs, Pu [17] linked reduced HE susceptibility in CrMnFeCoNi HEA to hydrogen-attenuated planar slip of dislocations. Conversely, Bertsch [101] observed hydrogen-induced increases in deformation band density and twinning in the same alloy. These inconsistent findings underscore the need for systematic investigations to elucidate hydrogen-dislocation interactions in HEAs, where lattice complexity and solute atom diversity likely exert critical influences.

Table 3 presents HE susceptibility data for HEAs with different phase compositions, including tensile strength (σ_b_), elongation (δ), hydrogen content, crystal structure, and references for both hydrogen-free and hydrogen-charged conditions. Table 1 demonstrates diverse mechanical responses to hydrogen charging across HEAs, with significant variations in hydrogen concentration and crystal structures. Notably, FCC-structured HEAs maintain high ductility even at elevated hydrogen levels, attributed to their inherent plastic deformation capacity, enabling coordinated strain accommodation to counteract hydrogen-induced embrittlement.

## 4. Design Strategies and Prospects for HE Resistance in High-Strength and High-Toughness HEAs

### 4.1. Design Ideas for Hydrogen Embrittlement-Resistant HEAs

Hydrogen-assisted cracking (HAC) in alloys in hydrogen environments differs from hydrogen-induced cracking (HIC) by its emphasis on the synergistic effect of applied stress and hydrogen. During HAC, crack tip failure continuously occurs, followed by sequential crack propagation under the influence of hydrogen. Notably, hydrogen-induced phase transformation mechanisms are one of the primary factors affecting hydrogen activation energy trapping during HAC. Phase transformations may create microstructures favorable for hydrogen adsorption and diffusion, such as specific pathways, porosity, or defects.

#### 4.1.1. Oxide Film Protection and Phase Structure Regulation

Strategies to mitigate hydrogen-induced failure include alloying element additions, microstructural modification, and oxide film formation. Oxide films, particularly those formed by alloying elements like Cu [106,107], reduce hydrogen absorption and minimize embrittlement risks. The thickness, uniformity, and composition of the oxide film are key factors in hydrogen barrier performance. The thickness of the oxide film directly influences its physical barrier effect: a sufficiently thick oxide film (e.g., aluminum anodized films with thicknesses ranging from 5 to 20 μm) can effectively block the diffusion pathways of hydrogen atoms. A uniform oxide film prevents the formation of local weak regions that could serve as channels for hydrogen permeation. The chemical composition of the oxide film determines its hydrogen permeability and structural stability.

For example, in a study of AlCoCrCuFeNi high-entropy alloy by Lou [108], the CuO/Cr_2_O_3_ composite oxide film formed on the surface reduced the hydrogen permeability from 8.5 × 10^−11^ mol/(m·s·Pa) of pure metal to 1.2 × 10^−12^ mol/(m·s·Pa). In this study, an inner Cr_2_O_3_ and outer CuO structure were formed, and corrosion hydrogen embrittlement tests in urban wastewater environments verified that the hydrogen embrittlement index was significantly reduced.

On the other hand, in the Cu-containing pipeline steel studied by Yoo J [109], the nano-sized Cu-rich phases provided favorable traps for hydrogen capture. The uniformly dispersed fine Cu-rich precipitates provided numerous sites for hydrogen distribution, which helped to avoid high local hydrogen enrichment and subsequent micro-regional hydrogen embrittlement. In the AlCoCrFeNi high-entropy alloy system, the addition of copper may also form Cu-rich nanoscale secondary phase precipitates, which are dispersed in the BCC matrix. Of course, this needs to be considered in combination with processing techniques.

Zhu [110] studied the effect of Al_2_O_3_ films on hydrogen diffusion behavior and found that, under normal pressure, Al_2_O_3_ exhibits a helical symmetric structure with a high hydrogen diffusion barrier (2.56 eV), leading to excellent hydrogen barrier performance. However, under high pressure (>100 GPa), the helical structure disappears, and the diffusion barrier drops to 0.24 eV, significantly reducing its hydrogen-blocking capability and making it unsuitable as a hydrogen diffusion barrier material. Cai [111] explored the relationship between micro-arc oxidation (MAO) films and HE sensitivity, revealing that the mechanism by which the film inhibits HE is primarily attributed to the suppression of hydrogen permeation and the additional compressive stress induced by the film. Jiang [112] investigated the effect of MAO films on hydrogen-induced local plastic deformation in 7050 aluminum alloy and measured that the stress corrosion cracking threshold (KISCC) of specimens with MAO films was 23.340 MPa·m^1^/^2^, compared to 16.934 MPa·m^1^/^2^ for specimens without MAO films. Additionally, the MAO film can reduce the hydrogen content at the crack tip of the aluminum alloy, thereby inhibiting hydrogen-induced local plastic deformation.

**Figure 26:**
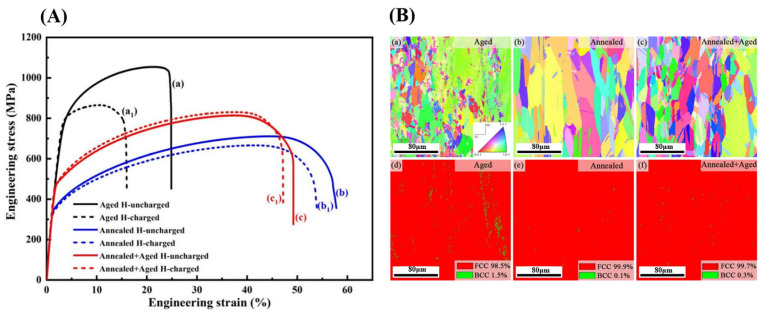
Engineering stress–strain curve (**A**) and EBSD characterization analysis (**B**). Engineering stress–strain curves of the (**a**,**a1**) aged, (**b**,**b1**) annealed, and (**c**,**c1**) annealed + aged Al_0.25_CoCrFeNi alloy samples without and with hydrogen charging [113]. (**b**) EBSD IPF images and phase maps of the (**a**,**d**) aged, (**b**,**e**) annealed, and (**c**,**f**) annealed + aged samples [113]. Reprinted from Ref. [113], Copyright 2024, Elsevier.

Extensive research has identified B2 phases as highly susceptible to embrittlement in hydrogen environments [16]. Therefore, material design should prioritize ductile phases capable of accommodating strain to counteract hydrogen-induced degradation and prevent irreversible hydrogen damage. Such strategies may involve optimizing B2 phase volume fractions through solution treatment to enhance hydrogen resistance. In their study, Liu [113] examined the effects of annealing on Al_0.25_CoCrFeNi high-entropy alloys. Their findings indicated that annealing led to the reduction of the B2 phase and the enhancement of resistance to hydrogen embrittlement. The annealing treatment (920 °C/4h) resulted in complete recrystallization of the samples, while the number of second phases, including the B2 phase, was dramatically reduced (the proportion of the BCC phase was reduced from 1.5% in the aged samples to 0.1%). The reduction of the second phase has been shown to reduce the risk of hydrogen-induced cracking and to result in a significant increase in the HE resistance of the annealed samples (plasticity loss of only 6.8%). Precipitation phase modulation and HE resistance were optimized by further annealing and aging at 920 °C for 4 h and 650 °C for 24 h, respectively. The treatment resulted in the formation of fine, dispersed B2 phase precipitates at the grain boundaries, replacing the coarse, aggregated second phases. The aggregation of hydrogen was found to be suppressed by these fine precipitated phases, which functioned as advantageous traps for hydrogen. This finding indicates that the susceptibility of HE can be further regulated through modulation of the tissues, as shown in Figure 26A,B.

#### 4.1.2. Nano-Precipitate Trap Design

In addition, nano-precipitates in HEAs have been proven to serve as hydrogen traps, offering the potential to further enhance HE resistance. Introducing nanoscale carbide NbC into the matrix is an effective method to improve HE resistance.

Fu [114] added Nb to the Fe_50_Mn_30_Co_10_Cr_10_C_0.5_ matrix, and the results showed that Nb induces the formation of nanoscale NbC precipitates and grain refinement strengthening. The NbC precipitates not only significantly improve the mechanical properties but also inhibit hydrogen diffusion and the formation of martensite (a microstructure detrimental to HE resistance). This is attributed to the strong hydrogen adsorption capacity of semi-coherent NbC precipitates. As confirmed in Shi’s work [115], density functional theory (DFT) calculations and experiments have demonstrated that the binding energy between the NbC/α-Fe semi-coherent interface and hydrogen reaches 0.80 eV, indicating that the NbC/α-Fe semi-coherent interface can adsorb a large amount of diffusive hydrogen, thereby achieving optimal HE resistance.

**Figure 27:**
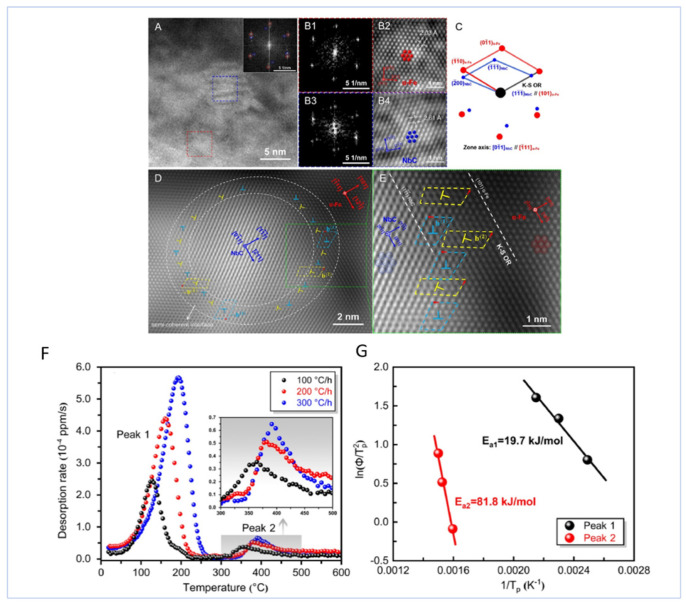
This figure shows the high-resolution transmission electron microscopy (HRTEM) observations along the [011]_nβ_C//[101]α−Fe direction at the NbC/α-Fe semi-coherent interface in Q&T-480 steel and the determination of the deep hydrogen trapping of NbC in the Q&T-480 steel [115]. (**A**) shows small NbC particles (FCC structure) inside the α-Fe matrix (BCC structure). The insets (FFT patterns) show a specific orientation relationship between NbC and α-Fe. (**B1**–**B4**) analyze selected areas to confirm the atomic structure of both phases along specific crystal directions. (**C**) shows they follow the Kurdjumov–Sachs (K-S) relationship, a type of semi-coherent interface where certain crystal planes and directions align closely. (**D**) highlights dense misfit dislocations (marked with symbols) that form to relieve stress from lattice mismatch (16.61%). These dislocations have specific Burgers vectors and help reduce interface strain. (**E**) enlarges part of the interface, showing that the dislocations are regularly spaced (~7.0 Å), matching calculations for stress relief. (**F**) TDS profiles at different heating rates, with Peak 2 shown in the enlarged inset (The arrows in the figure indicate local magnification). (**G**) Plot of ln(ϕ/Tp2) as a function of 1/T_p_ to obtain the activation energy for hydrogen desorption, E_a._ Reprinted from Ref. [115], Copyright 2024, Elsevier.

Figure 27 shows the atomic-scale structure and crystallographic orientation relationship of the NbC/α-Fe semi-coherent interface. A Kurdjumov–Sachs (K-S) orientation relationship between NbC and α-Fe was observed via high-resolution transmission electron microscopy (HRTEM). The hydrogen detachment behavior of a precharged hydrogen sample (Q&T-480 steel) was analyzed using thermal desorption spectroscopy (TDS) with different heating rates (100, 200, and 300 °C/h) and Kissinger’s formula to calculate the activation energy of hydrogen detachment was applied. The results show that the sample containing NbC nanoparticles exhibits two detachment peaks: Peak 1 (19.7 kJ/mol) corresponds to reversible traps (e.g., dislocations), and Peak 2 (81.8 kJ/mol) corresponds to deep hydrogen traps at the NbC/α-Fe semi-coherent lattice interface, indicating that NbC nanoparticles can effectively trap hydrogen atoms and decelerate hydrogen diffusion. This high-binding-energy hydrogen trap is more stable and tends to reduce the risk of hydrogen embrittlement, as shown in Figure 27F,G.

#### 4.1.3. Grain Boundary Strengthening

**Figure 28:**
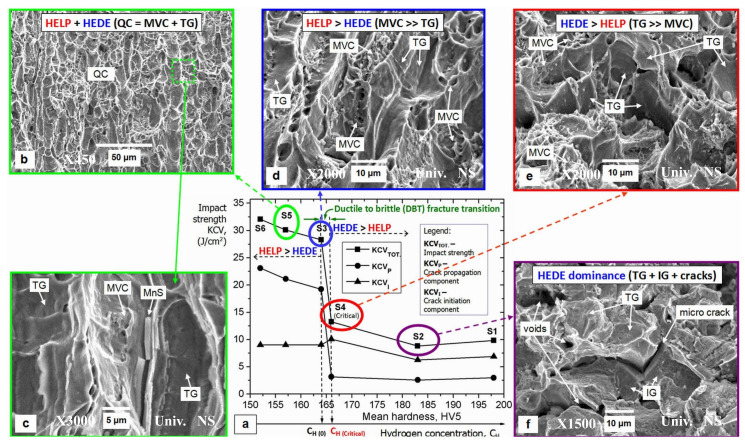
HELP + HEDE model in steels: (**a**) variation in impact strength (KCVTOT) and its crack propagation component (KCVP) and crack initiation component (KCVI) of Charpy specimens (S1–S6), as a function of the specimens’ hardness (hydrogen concentration); (**b**–**f**) SEM fractographs of the fracture surfaces of Charpy specimens (S2–S5) [116,117]. Reprinted from Ref. [116]. Copyright 2024, Elsevier.

Hydrogen has been observed to accumulate preferentially at defects such as grain boundaries and dislocations. These defects act as both trapping sites and fast diffusion pathways. While moderate hydrogen segregation at grain boundaries has been shown to impede catastrophic bulk embrittlement, excessive accumulation has been demonstrated to weaken grain boundary cohesion. The mechanisms in question are the HELP mechanism and the HEDE mechanism. In previous studies, intergranular cracking caused by the HEDE mechanism was often considered to be purely brittle. However, with the advancement of observational instruments, it was determined that the slip and tear marks that appear at specific fractures are not indicative of complete brittle fracture dominated by IG. This phenomenon can be attributed to the localized manifestation of greater plastic deformation, which is predominantly governed by the HELP mechanism at low concentrations. However, this assumption is predicated on the premise that the concentration at the grain boundaries must not exceed the critical hydrogen concentration. Furthermore, the hypothesis posits that the HELP mechanism, dominated by the HELP mechanism, delays crack initiation at the grain boundaries by increasing dislocation motion. The SEM morphology of the fully embrittled fracture due to HELP, HELP+HEDE, and HEDE is macroscopically interpreted in comparison to the different concentrations and further confirmed by the macro-mechanical properties of the HELP, HELP+HEDE, and HEDE by Milos B. Djukic [116] et al., as demonstrated in Figure 28. Moreover, this body of literature meticulously delineates the precise mechanism and expounds on the attendant effects [117]. Strategies to enhance grain boundary strength include grain refinement and microstructural homogenization. Grain refinement strengthening may optimize the mechanical properties of traditional alloys. Koyama M [118] achieved grain refinement of a CoCrFeMnNiHEA through rolling and annealing treatments. Although gaseous hydrogen charging confirmed improved resistance to hydrogen embrittlement, this process promoted the formation of a secondary σ phase—a well-known factor increasing hydrogen embrittlement sensitivity.

Furthermore, material design and processing should be executed in a manner that avoids the presence of sharp notches and effectively eliminates residual stresses that may be present after the welding process. Electroplating processes are known to introduce hydrogen. This issue can be mitigated through the application of heat treatment baking, which serves to remove any trapped hydrogen. This method is widely regarded as a standard practice to prevent hydrogen-related damage.

#### 4.1.4. Optimization of HEAs Fabrication Methods

Fabrication methods of HEAs have evolved significantly. Vacuum arc melting, a mature technique, produces dense surfaces with reduced hydrogen ingress risk; however, it may cause volatile element loss and lacks scalability. Miao [119] recently developed kilogram-scale AlCr_1.3_TiNi_2_ EHEA via electromagnetic levitation melting and direct casting, demonstrating stable high-temperature mechanical properties and advancing practical applications. EHEAs offer a promising platform for achieving high-strength-ductility combinations, leveraging their unique atomic mixing effects.

After material fabrication, internal residual stresses are often introduced, which can induce hydrogen-assisted cracking (HAC). Thus, hydrogen embrittlement caused by residual stresses cannot be overlooked. However, there is currently scarce research on the influence of residual stresses generated by HEA fabrication techniques.

The factors governing residual stress are complex and depend on different processing or post-processing methods. For example, shot peening introduces compressive residual stress, reaching a peak (approximately 1300 MPa) at 50 μm below the surface. If superimposed with high-amplitude tensile stresses during service, this leads to an increase in local net tensile stress, a reduction in the hydrogen diffusion barrier, and the promotion of hydrogen atom accumulation at defect sites such as grain boundaries and dislocation cores, forming critical hydrogen concentrations that induce crack initiation. The superposition of residual tensile stress and external stress decreases the material’s critical stress intensity factor for hydrogen embrittlement (KISCC). For instance, welding-induced residual tensile stress significantly reduces the KISCC of pipeline steels, while shot peening-induced compressive stress inhibits crack propagation [120]. This indicates that residual tensile stress is detrimental to HAC resistance.

In practical engineering applications, controlling load amplitude can reduce local net tensile stress and suppress hydrogen-induced crack initiation. For welded steel materials, post-weld heat treatment (PWHT) is recommended to relieve residual stress. For example, PWHT of high-strength pipeline steel (e.g., X80) at 600–650 °C (with isothermal holding) promotes hydrogen desorption and reduces residual tensile stress, resulting in a 30–50% reduction in weld-zone residual stress and a more than 50% decrease in hydrogen content.

### 4.2. Research Directions and Prospects for HE Resistance in HEAs

#### 4.2.1. Conclusions

(1) The concealment and delayed nature of hydrogen embrittlement (HE) pose significant challenges for material development. High-entropy alloys (HEAs), due to their multi-principal-element design, exhibit excellent hydrogen embrittlement resistance and comprehensive properties.
(2) Microstructurally, single-phase FCC (face-centered cubic) structures show superior HE resistance, while grain refinement may promote the formation of secondary phases (e.g., σ phase), increasing HE susceptibility.
(3) EHEAs can achieve high-strength and high-toughness HE-resistant alloys through microstructural regulation of phase composition.
(4) Additive manufacturing, alloying element addition (e.g., Cr, Mo, and Al), and heat treatment are effective strategies to optimize the HE resistance of HEAs.
(5) Future research should leverage machine learning modeling, cross-scale experiments, and environmentally friendly processing to advance HEA applications in the hydrogen energy field.
(6) In the mechanism analysis, we proposed the influence of key mechanisms, indicating that HEAs may face a situation where the HELP mechanism mediates HEDE; that is to say, the key lies in the role of hydrogen concentration, which determines the form of fracture.

#### 4.2.2. Future Research Directions

As emerging multi-principal-element alloys, HEAs exhibit distinct hydrogen solubility and diffusion behaviors compared to conventional alloys due to their unique compositional structure, including high-entropy effects, lattice distortion effects, and sluggish diffusion effects. Because of this, HEAs have significant potential in hydrogen energy applications. Current research focuses on HE resistance and the factors influencing it. However, the understanding of the mechanisms of hydrogen-induced failure remains limited. Future investigations should prioritize the following directions:

(1) Synergistic Mechanisms of HE and Corrosion

Atomistic-scale studies are needed to clarify hydrogen–atom interactions in HEA lattices, including hydrogen site occupation and its effects on electronic structures and chemical bonding. Additionally, HEA’s performance in hydrogen sulfide environments warrants further exploration. Under weakly acidic H_2_S conditions, material failure often results from combined HE and corrosion effects, which remain underexplored in HEAs.

(2) Multi-Mechanism Coupled Modeling

Integrated models incorporating multiple HE mechanisms, hydrogen internal pressure, surface energy reduction, lattice decohesion, localized plasticity, and strain-induced vacancy formation should be developed. Through machine learning and experimental validation, the relative contributions and interactions of these mechanisms under varying conditions, such as alloy composition, stress state, and hydrogen concentration, should be quantified. This will enable comprehensive prediction and interpretation of HE phenomena.

(3) Optimized HEA Composition and Microstructure Design

The current understanding of alloying element effects on HE susceptibility to develop novel HEA systems via high-throughput computational screening and experimental validation should be leveraged. Advanced processing techniques (additive manufacturing, powder metallurgy, thermomechanical treatment) to precisely control microstructural parameters, including grain size, phase composition, crystallographic orientation, and defect distribution should be explored.

## Figures and Tables

**Table 1:** Total concentration of deuterium atoms in four alloys [52]. Reprinted from Ref. [52]. Copyright 2024, Elsevier.

Material	Deuterium Charged Time (h)	Deuterium Concentration (wt.ppm)	Deuterium Desorption Peak (°C)
Ni	24	115.7	353/510
NiFe_20_	24	21.21	491
NiCoCr	24	82.05	485
NiCoCrFe	24	16.29	493
NiCoCrFeMn	6	46.51	495
NiCoCrFeMn	12	150.88	492
NiCoCrFeMn	24	275.35	495
Background	0	0.01	-

**Table 2:** Effective hydrogen diffusivities and trapped contents of HEA, SS 316L, and SS 304 using electrochemical hydrogen permeation testing [89]. Reused from Ref. [89]. Copyright 2024, Elsevier.

Specimens	D_eff_ (m^2^/s)	C_0_ (mol/m^3^)
CoCrFeMnNi	1.81 × 10^−11^	0.043
SS304	0.73 × 10^−11^	0.113
SS316L	1.31 × 10^−11^	0.048

**Table 3:** HE sensitivity of different high-entropy alloys (electrochemical hydrogen charging).

Material	*σ_b_*/MPa Un-Charge	*σ_b_*/MPa (Charge-H)	*δ*/% (Un-Charge)	*δ*/% (Charge-H)	Hydrogen Content (wt.ppm)	Crystal Structure	Reference
CoCrFeMnNi	730	730	58	46	5.65	FCC	[102]
(NiCoFe)_86_Al_7_Ti_7_	1324	1351	35.5	29.6	-	FCC + L1_2_	[50]
AlCoCrFeNi_2.1_	1007	959	19.4	15.6	9.26	FCC + B_2_	[82]
Ni_50_Cr_20_Co_15_Al_10_V_5_	1270	1197	24.2	17	26.72	FCC	[79]
FeCoCrNi	810	812	38	43	5.13	FCC	[103]
Co_30_Cr_10_Fe_10_Al_18_Ni_30_Mo_2_	1131	949.8	13.5	9	-	FCC+B2	[75]
Al_0.25_CoCrFeNi	578.8	622.1	63.7	70.2	-	FCC	[104]
Fe_50_Mn_30_Cr_10_Co_10_	700	739	57	50	2.48	FCC + HCP	[105]

## Data Availability

No new data were created or analyzed in this study. Data sharing is not applicable to this article.

## Abbreviations

The following abbreviations are used in this manuscript:

HE	Hydrogen embrittlement
HIC	Hydrogen-induced cracking
HAC	Hydrogen-assisted cracking
MEA	Medium-entropy alloy
*φ_HU_*	Reduction of area before hydrogen charging
*φ_HC_*	Reduction of area after hydrogen charging
*C_L(δ)_*	Rate of change of the subsurface concentration
*r_ad_*	Adsorption and Desorption Rates (Surface Processes)
*C_L(δ)_*	Subsurface Concentration Change Rate
*R_ads_*	Surface-to-Subsurface Adsorption Rate
*k_ads_*	Subsurface Adsorption Rate Constant
*k_des_*	Subsurface Desorption Rate Constant
*k_r_*	Desorption Rate Constant
*k_ad_*	Adsorption Rate Constant
*J_L_*	Bulk Diffusion Flux
*r_r_*	Surface Desorption Rate
*r_des_*	Subsurface-to-Surface Desorption Rate
*v*	Rate of change of surface coverage
*D_L_*	Bulk Diffusion Coefficient
*δ*	Subsurface Thickness
*C_L_*	Bulk Hydrogen Concentration
*σ_b_*	Ultimate tensile strength
*δ*	Percentage elongation after fracture
